# Exercise habits and glucose management among older adults with type 1 diabetes using insulin pumps

**DOI:** 10.1007/s00592-022-01858-3

**Published:** 2022-03-01

**Authors:** Anindita Chakrabarti, Andisheh Mohammad Alipoor, Thevia Ranjine Sandra Segaran, Spiros Fourlanos, Richard J. MacIsaac, Peter G. Colman, Sybil A. McAuley

**Affiliations:** 1 Department of Medicine, The University of Melbourne, Melbourne, Australia; 2 Department of Endocrinology and Diabetes, St Vincent’s Hospital Melbourne, Melbourne, Australia; 3 Department of Diabetes and Endocrinology, Royal Melbourne Hospital, Melbourne, Australia

**Keywords:** Older adults, Exercise, Insulin pump, Type 1 diabetes, Hypoglycaemia, Continuous glucose monitoring

## Introduction

Type 1 diabetes (T1D) glucose management is especially challenged by exercise, with greater glucose variability and risk of hypoglycaemia during and after physical activity [1]. With advancing age, adults with T1D may progressively experience additional challenges exercising, potentially relating to increasing prevalence of diabetes complications and other age-related comorbidities. Guidelines recommend older adults with T1D undertake at least 120–150 min of moderate-intensity aerobic activity and two 20-min sessions of resistance training weekly [2]. Evidence-based recommendations guide exercise-related T1D glucose management for paediatric and general adult populations; however, the needs of older adults have not been specifically addressed. We aimed to assess physical activity levels, and diabetes self-management practices in relation to exercise, among older adults with T1D using insulin pump therapy.

## Methods

We conducted a cross-sectional survey involving adults aged ≥ 60 years, with T1D for ≥ 10 years and using an insulin pump, at a tertiary hospital in Australia. Participants were recruited from the OldeR Adult Closed Loop (ORACL) trial [3]. After obtaining consent, data were collected at a single study visit (via telephone or videoconference) and from medical records. Clinical characteristics, diabetes history, diabetes self-management knowledge, and physical activity practices were assessed. Activity levels were quantified by the Yale Physical Activity Survey [4]. Exercise and T1D self-management practices, confidence, goals, and diabetes self-management information sources were assessed via a questionnaire developed for this study.

## Results

**Fig. 1:**
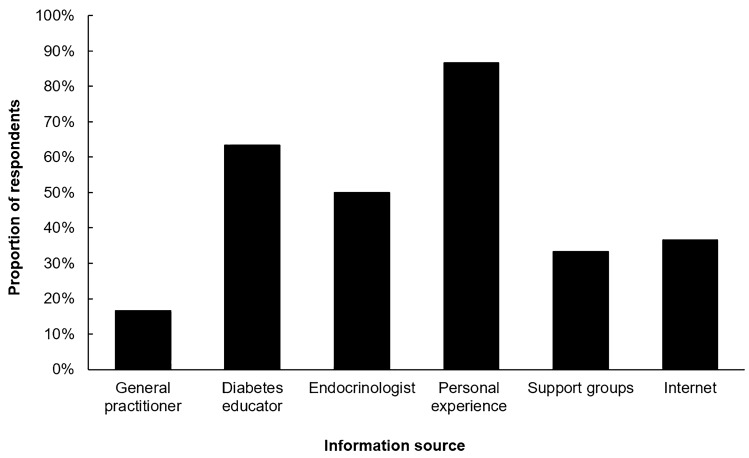
Reported sources of exercise-related diabetes management information

Thirty older adults (mean age 69 years [SD 5]; T1D duration 38 years [15]) participated. The group was physically active (exercise sessions on median 4 days/week [IQR 3, 6] of duration 60 min [40, 90]). Physical activity undertaken was mostly of light-to-moderate intensity (e.g. walking, gardening). Twenty-five participants (83%) were currently using real-time continuous glucose monitoring (CGM); seventeen (57%) were using a first-generation commercial closed-loop system with automated basal insulin delivery. All had real-time CGM experience. Their goals and types of physical activity varied, and their self-confidence in exercise-related diabetes self-management was high (Table 1). The commonest reported source of exercise-related diabetes management knowledge was personal experience, followed by advice from diabetes clinicians (Fig. 1). Concerns about glucose fluctuation influenced exercise participation for 21 participants (70%). For three participants (10%), fear of hypoglycaemia limited physical activity (two using real-time CGM and one using intermittently scanned CGM). Twenty-four participants (80%) always checked their glucose levels pre-exercise. Five participants (17%) routinely consumed bedtime snacks to avoid exercise-related overnight hypoglycaemia; however, four of those participants also routinely administered an insulin bolus dose with these snacks.

**Table 1:** Participant characteristics (*n* = 30)

Age (years)	69 (5)
Age range (years)	61 to 77
Women | men	19 (63%) | 11 (37%)
Type 1 diabetes duration (years)	38 (15)
Duration of insulin pump therapy (years)	10 (5, 14)
HbA_1c_ (%)	7.3 (0.5)
HbA_1c_ (mmol/mol)	56 (3)
Hypoglycaemia awareness	
Gold score	3 (2, 4)
Impaired awareness (Gold score ≥4)	10 (33%)
Current insulin delivery modality	Closed-loop therapy: 17 (57%)
	Manual insulin pump: 13 (43%)
Current glucose monitoring	Real-time CGM: 25 (83%)
	Intermittently scanned CGM: 1 (3%)
	Capillary blood glucose only: 4 (13%)
Total active time (hours per week)^a^	26 (15, 41)
Confidence in exercise-related diabetes self-management^b^	6 (5, 7)
Goals for engaging in exercise	Glucose management: 17 (57%)
	Fitness: 27 (90%)
	Weight loss: 18 (60%)
	Leisure: 21 (70%)
	Necessary daily activity: 13 (43%)
Type of exercise—most frequent and strenuous planned physical activity/exercise undertaken (in the past month)	Walking: 16 (53%)
	Gardening: 4 (13%)
	Gym class: 3 (10%)
	Cycling: 3 (10%)
	Pilates: 2 (7%)
	Woodwork: 1 (3%)
	Golf: 1 (3%)

Data presented are *n* (%), mean (SD), or median (IQR)

*CGM*: continuous glucose monitoring

^a^Total active time describes non-sedentary activity as defined by the Yale Physical Activity Survey (YPAS) which comprises a self-reported estimation of time spent engaged in non-sedentary activities including housework, gardening, shopping, meal preparation, caring responsibilities, and exercise (Dipietro L, et al. Med Sci Sports Exerc, 1993)

^b^Self-reported confidence score: ranging from 1 = Not confident at all; 4 = Partially, it still worries me; 7 = Very confident

All 30 participants reported that insulin pump therapy benefited their diabetes self-management around exercise; benefits noted were flexibility of insulin dosing, no injections, convenience, and the options to adjust or suspend basal insulin. Twenty-two participants (73%) stated that CGM had positive impacts on their glycaemic management around exercise; benefits included continuous glucose level and trend information, allowing for better adjustment of insulin dosing, and fewer exercise interruptions for capillary glucose testing. However, two participants (7%) considered CGM hindered their diabetes management around exercise due to additional blood glucose testing requirements (for CGM calibrations or verifying false low-glucose alerts).

Of the 17 closed-loop users, 11 reported automated insulin delivery improved their glucose management around exercise. The other six felt that closed-loop therapy did not confer exercise-related glucose benefits; the burdens described included experiencing hypoglycaemia despite using a temporary higher glucose target, delayed effects of insulin adjustment, and higher confidence with personally adjusting insulin dosing than the closed-loop algorithm. Two closed-loop users routinely implemented a temporary raised glucose target to avoid hypoglycaemia. Of the 13 participants using manual insulin pump dosing, four reduced their basal insulin rate before or during exercise (reductions ranging from 20 to 50%, starting 0 to 60 min before exercise); one participant routinely reduced their basal rate immediately after exercise.

## Discussion

Insulin pumps and CGM had positive impacts around exercise for most of this active group of older adults with long-duration T1D. The majority had high levels of self-confidence in managing their diabetes around exercise. Among the group, concern about glucose variability and fear of hypoglycaemia were barriers to participating in physical activity. Although the majority routinely checked their glucose levels pre-exercise, most did not consistently correctly implement other recommended strategies to avoid exercise-related glucose excursions.

Participants in this study were very active, spending a median 26 h/week engaged in physical activity. However, approximately half reported leisurely walking as their most strenuous activity, thus not reaching recommended exercise intensity [2].

Most closed-loop users found that closed loop benefitted their exercise-related glucose management, in keeping with research demonstrating that older age is not necessarily a barrier to embracing diabetes technology [3, 5]. The majority of study participants demonstrated a ‘trial-and-error’-style approach to exercise-related glucose management, highlighting potential roles for clinicians to actively engage in further education. Guidelines regarding glucose management around exercise support an individualised approach, including consideration of the intensity and duration of activity [2]. Individualisation of strategies recognising frailty, comorbidities, hypoglycaemia awareness, and current diabetes technology and therapeutics (particularly CGM) is paramount to support engagement in exercise with advancing age and T1D.

Study strengths include the detailed exploration of T1D self-management practices and the use of diabetes technology among an older cohort who are often excluded from studies. However, generalisability is limited by the small sample size and the highly-selected group; the findings should therefore be interpreted in this context. Nevertheless, even for this group of active, relatively healthy older adults with well-controlled T1D and familiarity with technology, significant barriers to exercising were noted. The broader population of older adults with T1D, many of whom face additional health-related challenges than this group, could be expected to face even greater barriers to exercise.

This study provides insight into potential benefits and disadvantages of insulin pumps and CGM for exercise among older adults with T1D. Our observations imply greater use of technologies for older adults with T1D may assist with reducing barriers to exercise through safer and more optimal exercise-related glucose management. Future research specifically including older adults is required to optimise the potential benefits of diabetes therapeutic technology for older adults undertaking exercise.

## Data Availability

The data generated will be shared by the corresponding author upon reasonable request.

## References

[1] McAuley SA, Horsburgh JC, Ward GM (2016). Insulin pump basal adjustment for exercise in type 1 diabetes: a randomised crossover study. Diabetologia.

[2] Sinclair AJ, Dunning T, Dhatariya K (2020). Clinical guidelines for type 1 diabetes mellitus with an emphasis on older adults: an executive summary. Diabet Med.

[3] McAuley SA, Trawley S, Vogrin S (2021). Closed-loop insulin delivery versus sensor-augmented pump therapy in older adults with type 1 diabetes (ORACL): a randomized, crossover trial. Diabet Care.

[4] Dipietro L, Caspersen CJ, Ostfeld AM, Nadel ER (1993). A survey for assessing physical activity among older adults. Med Sci Sports Exerc.

[5] Pratley RE, Kanapka LG, Rickels MR (2020). Effect of continuous glucose monitoring on hypoglycemia in older adults with type 1 diabetes. JAMA.

